# Analysis of the Status of the Cutaneous Endogenous and Exogenous Antioxidative System of Smokers and the Short-Term Effect of Defined Smoking Thereon

**DOI:** 10.3390/antiox9060537

**Published:** 2020-06-19

**Authors:** Silke B. Lohan, Karl Bühring, Anna-Christina Lauer, Annette Friedrich, Jürgen Lademann, Annette Buss, Robert Sabat, Kerstin Wolk, Martina C. Meinke

**Affiliations:** 1 Center of Experimental and Applied Cutaneous Physiology, Department of Dermatology, Venereology and Allergology, Charité—Universitätsmedizin Berlin, Charitéplatz 1 (corporate member of Freie Universität Berlin, Humboldt-Universität zu Berlin, and Berlin Institute of Health), 10117 Berlin, Germany; silke.lohan@charite.de (S.B.L.); karl.buehring@gmx.de (K.B.); annaclauer@yahoo.de (A.-C.L.); Annette.u.friedrich@gmail.com (A.F.); juergen.lademann@charite.de (J.L.); 2 Psoriasis Research and Treatment Center, Department of Dermatology, Venereology and Allergology and Institute of Medical Immunology, Charité—Universitätsmedizin Berlin, Charitéplatz 1, 10117 Berlin, Germany; annette.buss@charite.de (A.B.); Robert.Sabat@charite.de (R.S.); kerstin.wolk@charite.de (K.W.); 3 BIH Center for Regenerative Therapies, Charité—Universitätsmedizin Berlin, Augustenburger Platz 1, 13353 Berlin, Germany

**Keywords:** cutaneous redox system, antioxidants, electron paramagnetic resonance (EPR) spectroscopy, quantitative real-time PCR, resonance Raman spectroscopy

## Abstract

The daily consumption of tobacco products leads to a boost in free radical production in tissues, promoting the risk for malignancies, metabolic alterations and chronic-inflammatory diseases. This study aimed to broaden the knowledge of the status of the antioxidative (AO) system in the skin, compared to the blood, of healthy appearing smokers. Both, the basic status compared to non-smokers and the short-term impact of controlled cigarette consumption in smokers were analyzed. Our study showed that the basic level of the AO system of smokers significantly differed from that of non-smokers. As determined by resonant Raman spectroscopy (RRS), the levels of exogenous AOs were decreased in both, the skin, in vivo (β-carotene and lycopene), and blood plasma (β-carotene only). In contrast, the levels of glutathione (GSH), the prototypical endogenous AO, which were analyzed by fluorimetric assays in cutaneous tape strips and blood plasma, were increased in the skin, although unchanged in the blood of smokers. Elevated cutaneous GSH levels were reflected by an elevated overall radical scavenging activity in the skin, as quantified by non-invasive electron paramagnetic resonance (EPR) spectroscopy. Analysis of the expression of selected stress-associated genes in blood immune cells by quantitative RT-PCR in subgroups of non-smokers and smokers additionally demonstrated the downregulation of AKR1C2 in smokers, and its negative correlation with blood plasma levels of the protective immune mediator interleukin-22, assessed by the ELISA technique. Controlled cigarette consumption did not alter exogenous or endogenous AOs in the skin of smokers, but decreased lycopene levels in blood plasma. Moreover, there was a decline in blood IL-22 levels, while no relevant response of blood cell gene expressions was found after the considered short time. Our data therefore demonstrate a strengthened endogenous AO status in the skin of smokers, which may indicate a long-term adaptation to chronic oxidative stress in this specific organ. While this effect was not clearly visible in the blood, this compartment seems to be useful as an immediate indicator of the body’s AO consumption. Moreover, decreased levels of AKR1C2, which we show for the first time to be expressed in immune cells, may be a candidate marker for long-term smoking. In addition, this study demonstrates that the rate constant of a spin probe decline determined by EPR spectroscopy mainly represents the endogenous AO status of a tissue.

## 1. Introduction

Physical and mental stress, and also certain habits such as regular cigarette smoking, promote the generation of free radicals, like reactive oxygen species (ROS) in our cells [1,2]. The consequences of cellular ROS accumulation include an increased oxidation of cell components, such as lipid membranes, proteins and nucleic acids, leading to cell and tissue damages [3]. The habit “smoking” is one of the main causes of oxidative stress development in the body. In fact, cigarettes contain over 600 different ingredients, creating more than 7000 chemicals when burned, and inducing up to 10^15^–10^17^ free radicals in the aerosol per puff during cigarette smoking [4]. Therefore, it is not surprising that the consumption of cigarettes and other tobacco products is associated with physiological disorders, such as pulmonary diseases, cardiovascular dysfunction, reproductive and developmental effects and insulin resistance [5]. ROS are also significantly involved in the development of inflammatory skin diseases, skin cancer and premature skin aging [6,7].

In order to minimize oxidative stress, our body possesses an antioxidative defense system to neutralize free radicals. Antioxidants (AOs) are chemical compounds, which remove or detoxify free radicals and/or slow down undesirable oxidative processes at the cellular components [8,9]. Endogenous AOs and those ingested with food (exogenous AOs) can be distinguished. At stress-free conditions (basic state), these compounds are adequately present, keeping the level of ROS at non-toxic levels within the cells (so called redox homeostasis). Exogenous AOs include, among others, vitamin C and E, as well as carotenoids. A combination of vitamins C and E appears to have a certain protective effect on smoke-induced oxidative stress, which is probably based on its radical-scavenging effect [10,11]. In general, β-carotene has been demonstrated to possess AO activity, thus, the risk of diseases associated with increased ROS levels might be reduced by its uptake in physiological doses in the form of fruits and vegetables or nutritional supplements [12]. Cutaneous carotenoids are often considered as marker substances for the total AO state of the human skin [13]. It should be noted, however, that the intake of β-carotene as a single component in doses exceeding the physiological needs was shown to be counterproductive by smokers, because this vitamin A precursor seems to increase lung cancer incidences [14,15].

Endogenous AOs are generated by our body itself and can be subdivided into enzymatic and non-enzymatic ones. The tripeptide glutathione (GSH) in its reduced form is the most abundant non-enzymatic antioxidant in cells, scavenging ROS. It is water-soluble and maintains the reducing conditions by getting oxidized itself (GSSG). The NADPH-dependent glutathione reductase regenerates GSSG to GSH [16,17]. Interestingly, Bhatnagar A et al. [18] demonstrated increased blood levels of GSH, as well as of superoxide dismutase, an enzymatic AO, and a reduction of lipid peroxidation processes in non-smokers after 3 months of lifestyle change, with more physical activity and a balanced diet. The authors suggest that moderate stress, like a reasonable amount of physical activity, can activate certain adaptation mechanisms of the metabolism, leading to a strengthened endogenous AO [18].

Smoking primarily causes oxidation of GSH in bronchial epithelial cells, reflecting a reduction of the total AO capacity in the cells [19]. On the other hand, cellular studies presented by Altraja S et al. suggested the existence of compensatory mechanisms that lead to increased GSH levels after long-term exposition to cigarette smoke condensate [20].

In our study, the AO system of smokers versus non-smokers was assessed using different spectroscopic and molecular biological methods. Two questions were asked: first, does the basic AO status differ between smokers and non-smokers (long-term effect of smoking)? Second, is there a short-term effect on the basic AO status after defined smoking?

Analyses were performed in the skin and, for comparison, in the blood plasma of non-, moderate and strong smokers. Moreover, gene expression analyses were performed from blood serum of selected molecules known to be involved in the maintenance of the redox balance and induced by the protective cytokine interleukin-22.

## 2. Materials and Methods

### 2.1. Subjects and Study Design

A total of 85 non-smokers (nSm; female 34/male 51, age structure: 31.5 ± 8.8 years), 18 moderate smokers (mSm; female 7/male 11, age structure: 29.2 ± 6.0 years), and 51 severe smokers (sSm; female22/male 29, age structure: 32.6 ± 7.6 years), were enrolled in our study. mSm were defined by consumption of <10 cigarettes/day, sSm were defined by consumption of ≥10 cigarettes/day. Participants were included in 4 different substudies (substudies A–D). An overview of the substudies with number of participants, gender distribution and smoking status is given in Table 1. The volunteers for analyzing the gene expression profile of stress-associated genes (study D) were a subgroup of study C. The volunteers for analyzing the gene expression profile of stress-associated genes (substudy D) were a subgroup of study C that was randomly selected. Since this was a pilot study, only 5 subjects were examined.

For a comparison of the general AO status of smokers versus non-smokers (substudies A, B and C), the time of the last cigarette consumption by the smokers was not controlled. The investigated time point is called “basic antioxidant status (time: 0 h)”. For investigating the short-term effect of defined smoking by study participants defined as severe smokers (sSm) (substudies C and D), reduced cohorts were investigated. These cohorts had not smoked for at least 2 h before the study start. Defined smoking included the consumption of 2 cigarettes at a time interval of 15 min in the fresh air. nSm (control group) also spent 15 min in the fresh air without any cigarette consumption. In order to minimize additional stress induced by other parameters (physical activity), the way to the fresh air (and back) was taken by a lift. Before further measurements were performed, a subsequent acclimatization was allowed. Re-measurements of the cutaneous antioxidant status were performed 4 h after defined cigarette consumption (sSm)/air exposure (nSm). For substudy C and D, blood samples were taken at 0 h and 4 h.

For defined cigarette consumption, the cigarette brand “Gauloises Blond red” (Imperial Tobacco, ingredients: tar: 7 mg, nicotine: 0.6 mg, carbon monoxide: 9 mg), was blindly used, which corresponds to the standard cigarette 2R4F [21].

Since, in substudy A, moderate smokers delivered measured values lying between those detected for nSm and sSm, mSm were not included in the subsequently performed pilot substudies B to D.

In addition, the volunteers completed a questionnaire, in order to collect data about nutrition, sport activities, sun exposure, mental stress and other habits.

All measurements were performed during the winter months and skin sites for analysis were chosen in minimally exposed body areas, thus, an influence by UV radiation could be excluded, and was also confirmed by the questionnaire by all volunteers.

All studies were approved by the Ethics Committee of the Charité — Universitätsmedizin Berlin, in accordance with the Declaration of Helsinki principles, as revised in 2000 (project identification code: EA1/211/13). All volunteers gave their informed written consent.

### 2.2. Determination of the Cutaneous Radical Scavenging Capacity by Electron Paramagnetic Resonance (EPR) Spectroscopy

As a measure of the antioxidant status of the skin, the radical scavenging activity was measured. To determine the radical scavenging activity of the skin of study participants by in vivo electron paramagnetic resonance (EPR) spectroscopy, the rate constant of a semi-stable radical (spin probe) was determined. For this, the spin probe TEMPO (2,2,6,6-tetramethylpiperidine-1-oxyl, 98%), purchased from (Sigma Aldrich, Steinheim, Germany), was applied onto the inner forearm of a volunteer.

**Figure 1:**
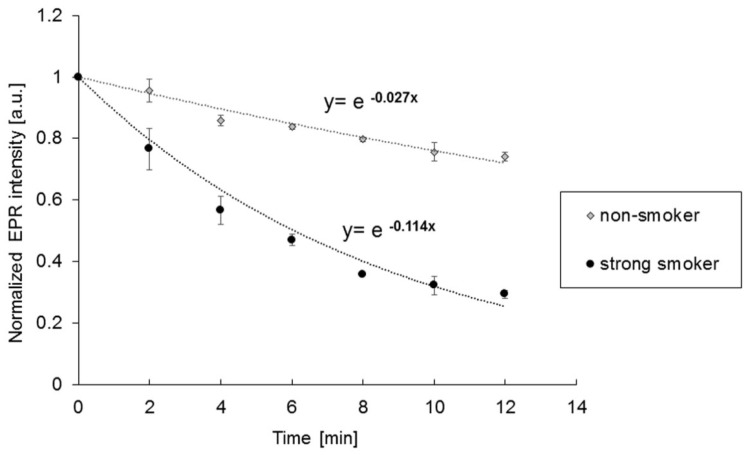
Exemplary electron paramagnetic resonance (EPR) signal decay (dots, triangle) over time from a non-smoker (nSm) and a strong smoker (sSm) for determining the basic antioxidant status (substudy A) and the respective fit by the simple exponential decay (dotted lines).

The antioxidants in the skin react with the spin probe TEMPO, and reduce it to the corresponding EPR silent hydroxylamine. The decay of the EPR signal intensity can be expressed as a simple exponential function I = I exp(−kt), where k is the rate constant. A high rate constant represents a high antioxidant status of the skin (Figure 1).

In brief, a 30 mM TEMPO solution (water/ethanol, 1:1) was prepared. Measurements were performed in vivo up to 4 times on the same inner forearm, to prevent fluctuations >20%. The skin was prepared by removing terminal hairs with scissors and cleaning with an ethanol-soaked cotton pad. 50 μL of the TEMPO solution was applied onto a filter paper (Ø 12 mm; SmartPractice Europe GmbH, Barsbüttel, Germany) and occluded with a Finn Chamber (SmartPractice Europe GmbH, Barsbüttel, Germany) for 10 min. After removing excess liquid, the forearm was placed on a rail in the EPR spectrometer and lifted to the measuring coil. The skin and surface coil remained separated by a thin cover glass (Menzel-Gläser, Braunschweig, Germany). During the measurement, the arm was fixed so that no movements could interfere with the EPR measurements. The decrease of the spin probe level was monitored over time, using an L-Band EPR spectrometer (LBM MT 03, Magnettech Berlin, Germany) with the following magnetic parameters: microwave frequency (1.3 GHz), central magnetic field (46 mT), sweep width (8 mT), sweep time (10 s) and modulation amplitude (0.15 mT). The forearm of a volunteer was measured for 12 min, 4 scans/min were recorded. In order to increase the signal-to-noise ratio, the mean spectrum of 8 scans was evaluated. All measured values were normalized to the first measured value of the respective measurement.

Since the obtained EPR in vivo-spectra are limited in their expression, a simple exponential function was used for evaluation to avoid an overinterpretation of the spectra. This is an approximation, but has been already applied in several other studies [22,23]. An increased concentration of antioxidants leads to a stronger drop in the curve.

### 2.3. Blood Sample Preparation

Blood plasma was separated from EDTA blood by centrifugation for 15 min at 1200 g and stored at −80 °C until use. Blood serum was separated from blood, coagulated for 30 min, by centrifugation for 10 min at 1000 g and was stored at −80 °C until use. Peripheral blood mononuclear cells were prepared from citrated blood by density gradient centrifugation, as previously described [24], resuspended in lysis solution from Invisorb RNA kit II (Invitek/Strated Molecular) and stored at −80 °C until messenger RNA analysis.

### 2.4. Determination of Carotenoids (β-Carotene and Lycopene) in Skin and Blood by Resonance Raman Spectroscopy (RRS)

**Figure 2:**
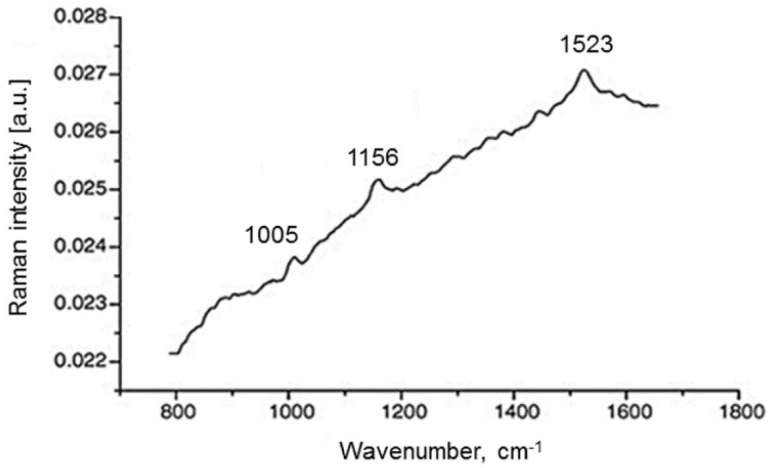
Exemplary Raman spectrum of beta-carotene and lycopene obtained in the human skin measured in vivo under 514.5 nm argon laser excitation. Raman lines at 1523 cm^−1^ were used for calculation of the beta-carotene or lycopene level respectively [25].

To determine the exogenous AOs β-carotene and lycopene in skin, a prototype of a resonance Raman spectroscopy (RRS) system produced in house was used. This RRS technique has been applied by Darvin et al. in numerous studies [25,26]. An argon laser was used: at a wavelength of 514 nm, predominantly the lycopene is excited, while the β-carotene is excited at 488 nm [25] (Figure 2).

Figure 2 shows a typical spectrum obtained in human skin with an excitation wavelength at 514.5 nm. A skin spectrum excited at 488 nm behaves similarly. After deducting the fluorescence signal of other biologic molecules, the three Raman bands of the carotenoids can be evaluated.

Raman lines located at 1005 cm^−1^, 1156 cm^−1^ and 1523 cm^−1^ each originate from the oscillations of the methyl groups, from carbon–carbon single bond and carbon–carbon double-bond stretch vibrations of the conjugated backbone.

The pronounced Raman line at 1523 cm^−1^, which exists at both 488 nm and 514 nm excitation, was used to detect both carotenoids, depending on whether the skin was excited, at 488 nm or 514 nm, respectively. A shift of 31.1 nm between these Raman lines is given. The Ar-laser light is focused on a skin area. The scattered light was collected by a lens and guided via a fiber cable into a spectrograph, which was connected to a charge-coupled device (CCD) camera and a laptop. The results are averaged from three measurements and displayed in arbitrary units. The non-invasive skin measurements were performed only on the right palm of study participants, as previously described, since the measured values for carotenoids are reflected in both limbs in a comparative way [25,27]. The non-invasive skin measurements were performed only on the right palm of study participants, as previously described, since the measured values for carotenoids are reflected in both limbs in a comparative way.

It has been shown that systemically administered carotenoids are stored in subcutaneous fatty tissue, and then slowly released to the skin surface via sweat and sebum. Thus, the epidermis on the ball of the thumb represents the best measuring point, as it has a thickness of at least 200 µm, preventing the laser radiation from reaching the blood vessels, and their carotenoid content affects the skin measurements; furthermore, the sun exposure is low at this location.

In total, 5 measurements per participants were taken, and the values were averaged subsequently.

In blood plasma, RRS measurements were performed using quartz glass cuvettes, as described previously [28]. Here, three measurements per blood sample were performed and then averaged.

### 2.5. Determination of Glutathione (GSH) in Blood and Skin

Glutathione was analyzed as representative for endogenous AOs. For glutathione detection in blood, blood plasma samples were purified by using the ReadiUse™ TCA Deproteinization Sample Preparation Kit (AAT Bioquest, Inc., Sunnyvale, CA, USA). GSH quantification was then performed by Amplite™ Fluorimetric Glutathione GSH/GSSG Ratio Assay Kit *Green Fluorescence* from AAT Bioquest, Inc. (Sunnyvale, CA, USA), according to the manufacturer’s instructions.

For GSH quantification in skin, tape stripping of the inside of the forearm was performed. The determination of glutathione from these adhesive films was based on previously described protocols [29,30,31]. Four adhesive tapes with a diameter of 22 mm (3 M, BlenddermTM, 1525-2 Neuss, Germany) were taken from the inside of the forearm; the adhesive tape was pressed onto the skin by using a roller to stretch the skin surface (10 times). The first tape was discarded; the three following tapes were immediately treated with liquid nitrogen, and then stored at −80 °C until further processing. The skin material was removed from the adhesive tapes by ultrasound (Sonorex Super, BANDELIN electronic GmbH & Co. KG, Berlin, Germany) in 800 µL of 0.05 M potassium phosphate buffer (1mM EDTA, pH 7.5–8.0). Then, 10 min ultrasound at 4 °C per tape was applied. After sonification of the first tape of the tape set, it was carefully removed from the tube, and the next tape was put into the same buffer solution followed by the next sonification process. This procedure was performed for the whole tape set of one volunteer. To remove insoluble material, the obtained solution was centrifuged at 16,000× *g* for 5 min at 4 °C (UNIVERSAL 320R (Hettich AG, Bäch, Switzerland). A total of 120 µL of the homogenized sample was transferred into a ViewPlate-96 Black (Perkin Elmer LAS, Rodgau Jügesheim, Germany) and mixed with 12.5 µL o-Phthalaldehyd/Methanol solution (P0532-50 mL, Sigma Aldrich, Steinheim, Germany). After 10 min incubation time at room temperature, GSH was measured in a plate reader (Enspire, Perkin Elmer, Rodgau Jügesheim, Germany), using an excitation wavelength at 350 nm and detection wavelength at 420 nm.

The GSH concentration was related to the protein content of the respective sample, determined by using Pierce Protein Reagent Assay BCA Kit according to the manufacturers‘ instructions (Thermo Fisher Scientific, Waltham, MA, USA #23227).

### 2.6. Messenger RNA Analysis in Peripheral Blood Mononuclear Cells

Isolation of total cellular RNA from freshly isolated blood cells was done by TRIzol^®^ Reagent (ThemoFisher Scientific, Darmstadt, Germany), according to the manufacturers protocol, and checked for quantity and quality by the RNA 6000 Nano LabChip Kit II, using the Bioanalyzer 2100 device (Agilent Technologies, Santa Clara (CA), USA). In the obtained RNA samples, the contained messenger-RNA fraction was reverse transcribed into complementary DNA (cDNA) using pd(T)18 primers, as described previously [32]. Afterwards, cDNA quantification was done by real-time (TaqMan^TM^, ThemoFisher Scientific, Darmstadt, Germany) polymerase chain reaction (RT-PCR), using the StepOne plus instrument, the Maxima Probe/ROX qPCR Master Mix, and ready-to-use detection assays (ThermoFisher Scientific, Darmstadt, Germany). In addition to the PCR primers, detection assays contain an oligonucleotide probe, in which a fluorophore is attached to the 5’-end, and a quencher at the 3’-end, allowing quenching of the fluorescence emitted by the excited fluorophore, as long as the probe is intact. When the probe hybridizes to the target cDNA sequence during PCR-based amplification steps, the 5’–3’ exonuclease activity of the DNA-synthesizing enzyme (Taq polymerase) cleaves the probe, to allow fluorophore-based quantification of the stepwise amplification, as a measure of the initial cDNA concentration in the sample. The following oxidative stress-associated genes were selected for the RT-qPCR examinations, because they are all involved in maintaining the redox balance: nuclear factor, erythroid 2 like 2 (NFE2L2, also known as NRF2), superoxide dismutase 2 (SOD2), transaldolase 1 (TALDO1) and lipocalin 2 (LCN2), aldo-keto reductase family 1, member C2 (AKR1C2). Analysis of the house-keeping gene hypoxanthine-guanine phosphoribosyltransferase (HPRT) was included to normalize expressions. Only changes of >1.5 fold were considered as being relevant.

### 2.7. Quantification of Interleukin-22 in Blood Serum

Levels of the protective interleukin-22 were quantified in blood serum by Quantikine^TM^ ELISA from R&D Systems/Biotechne (Minneapolis, MN, USA).

### 2.8. Data Analysis and Statistical Evaluation

Data were evaluated for statistical relevance using SPSS for Windows (SPSS Inc., Chicago, IL, USA). Evaluation was based on the Mann-Whitney U-test for non-related samples and Wilcoxon matched-pairs signed-rank test for related data. Correlations between parameters were analyzed using Spearman’s correlation analysis. *p*-values ≤ 0.05 were considered as significant.

## 3. Results

### 3.1. Basic AO Status (0 h)

In order to determine the basic AO status in the skin for nSm, mSm and sSm, different non-invasive spectroscopic approaches and molecular biological methods were performed: the tissue radical scavenging capacity was assessed by the absolute rate constant of the decay of the spin probe TEMPO, monitored by EPR. The GSH concentration was quantified in the upper skin layer (stratum corneum), using a fluorimetric assay sampled by tape stripping. The exogenous cutaneous carotenoids β-carotene and lycopene were determined by RRS.

**Figure 3:**
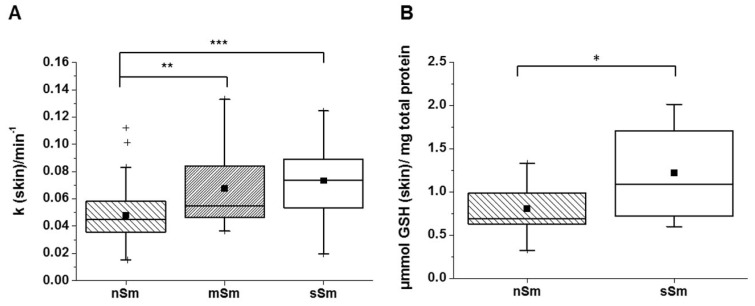
Basic level (time 0 h) of the antioxidant status in skin for non-smokers (nSm, *n* = 57), moderate (mSm, *n* = 18, < 10 cig./day) and strong smokers (sSm, *n* = 23, ≥10 cig./day), (**A**) absolute rate constant of radical scavenging capacity, as measured by non-invasive EPR spectroscopy; (**B**) glutathione (GSH) concentration for nSm (*n* = 13) and sSm (*n* = 13), as measured in tape-stripped stratum corneum by fluorimetric assay. The figure contains the results of study A, B and C (basic level (time 0 h), cutaneous antioxidant status); boxplot chart with percentile 25, 75, median (black line), mean (black square) and outlier (black cross), * *p* ≤ 0.05, ** *p* ≤ 0.01, *** *p* ≤ 0.001.

In Figure 3A, the cutaneous rate constants of the decay of the EPR signal of nSm, mSm and sSm are illustrated. The higher the rate constant, the higher the AO status of the skin. The sSm and also the mSm showed significantly elevated rate constants compared to nSm. No difference was detected between the two groups of smokers.

The analysis of the basic GSH level in the skin of sSm versus nSm (Figure 3B) revealed significant differences. On average, sSm showed a 1.5 times higher GSH concentration compared to nSm.

**Figure 4:**
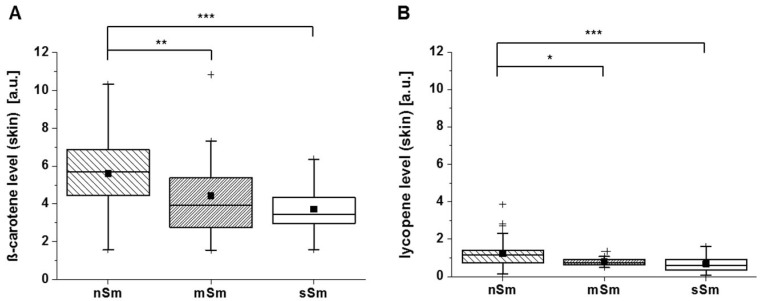
Basic level (time 0 h) of cutaneous carotenoids in skin for non-smokers (nSm, *n* = 57), moderate (mSm, *n* = 18, < 10 cig./day) and strong smokers (sSm, *n* = 23, ≥ 10 cig./day) analyzed by resonant Raman spectroscopy (RRS). The figure contains the results of study A and B (basic level (time 0 h), β-carotene, lycopene), (**A**) ß-carotene level [a.u.] (**B**) Lycopene level [a.u.]; boxplot chart with percentile 25.75, median (black line), mean (black square) and outlier (black cross), * *p* ≤ 0.05, ** *p* ≤ 0.01, *** *p* ≤ 0.001.

The spin probe TEMPO does not react with carotenoids, like β-carotene or lycopene [22]. As shown in Figure 4, both smoker groups, mSm and sSm, displayed significantly lower carotene levels in comparison to the nSm (Figure 4A). A similar pattern was detected for the lycopene values (Figure 4B).

These data suggest that the basic radical scavenging activity of the skin, which seems to be basically represented by the endogenous AO GSH (Figure 3B), is increased in smokers, suggesting a protecting adaptive mechanism induced by long-term smoking. In contrast, the levels of carotenoids as a measure of exogenous AOs were decreased in smokers (Figure 4), suggesting their first-line consumption in situations of permanently increased oxidative stress.

In the next step, we asked whether the pattern of the AO status detected in the skin might also be reflected by that in the blood plasma of study participants.

**Figure 5:**
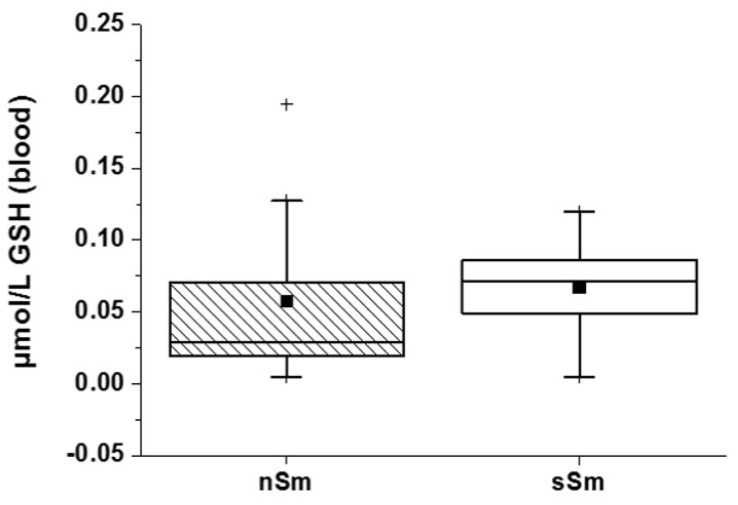
Basic status (time 0 h) of GSH concentration in blood plasma between non-smokers (nSm) and strong smokers (sSm, ≥ 10 cigarettes/day), (*n* = 10 each), as measured by fluorimetric assay). Boxplot chart with percentile 25, 75, median (black line), mean (black square) and outlier (black cross).

In blood, the GSH concentration was comparable between both groups (nSm vs. sSm) (Figure 5). At baseline, the smokers showed no active consumption of GSH in blood, concluding that no acute smoke stress is given, and their redox system is in equilibrium for their conditions.

**Figure 6:**
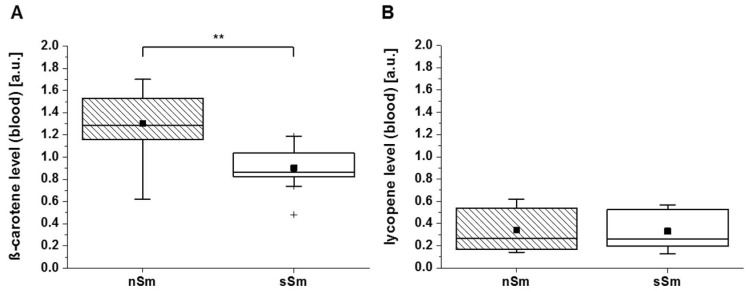
Basic status (time 0 h, study 2) of the β-carotene (**A**) and lycopene (**B**) level [a.u.] in blood plasma for non-smokers (nSm, *n* = 10) and strong-smokers (sSm, *n* = 10, ≥ 10 cigarettes/day), measured by RRS spectroscopy. Boxplot chart with percentile 25, 75, median (black line), mean (black square) and outlier (black cross), ** *p* ≤ 0.01.

As demonstrated in Figure 6A, a similar trend for the basic level of β-carotene in blood was found. For the lycopene level in blood, no differences were measured (Figure 6B). In general, the difference between nSm and sSm for both carotenoid levels were less pronounced in blood vs. skin.

A recent study stated elevated blood levels of the immune mediator interleukin-22 in healthy appearing long-term severe smokers [33]. Elevated blood levels of this protective cytokine indicate the activation of specific immune cells in tissues [34]. In our study, a trend of interleukin-22 levels increasing with the number of cigarettes consumed daily was observed (data not shown).

### 3.2. AO Status in Response to Defined Smoking

Subsequently, the short-term influence of a definite tobacco consumption on the AO status of smokers was examined. Therefore, sSm smoked two cigarettes in a time interval of 15 min in the fresh air; nSm (control group) also spent 15 min in the fresh air without any cigarette consumption (on ethical grounds). The AO status (skin/blood plasma) was determined after 4 h, and was compared to the basic level (time 0 h). The cutaneous AO status was measured by EPR, carotenoid levels were determined by RRS in skin and blood plasma. In addition, blood plasma was investigated for the redox system GSH.

**Figure 7:**
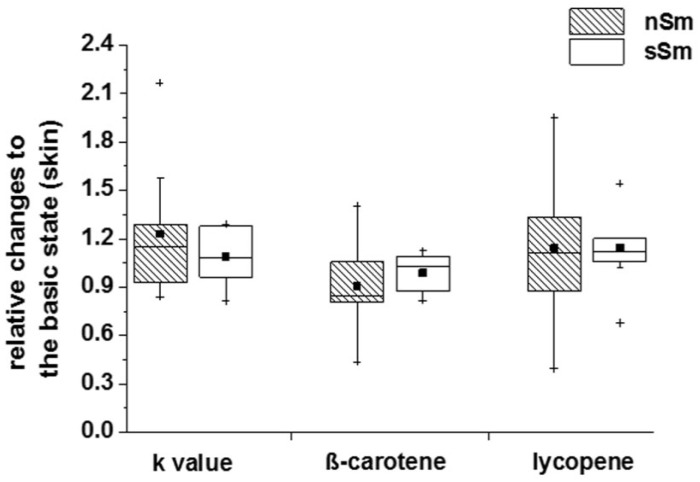
Impact of defined cigarette consumption on the cutaneous antioxidative (AO) status. sSm (*n* = 10) were allowed to smoke two cigarettes in a time interval of 15 min (sSm), while, for the same time, nSm (*n* = 10) were subjected to fresh air consumption. Cutaneous measurements of the rate constant of the spin probe TEMPO and the carotinoid levels were done by EPR spectroscopy and RRS, respectively, 4 h after smoking. Data relative to the basic level (time 0 h) are illustrated by boxplot charts, with percentiles 25 and 75, median (black line), mean (black square) and outliers (black cross).

Investigations of nSm and sSm 4 h after cigarette smoking/fresh air consumption, respectively, reveal no significant changes in the rate constants, cutaneous β-carotene level or lycopene level (Figure 7). In each case, a similar distribution pattern to the basic status (time 0 h) between the individual groups could be determined.

**Figure 8:**
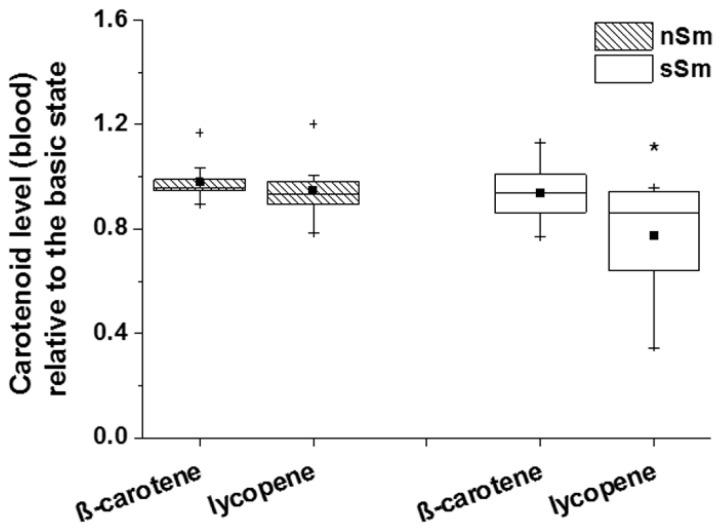
Impact of defined cigarette consumption on blood plasma levels of exogenous AOs. sSm (*n* = 10) were allowed to smoke two cigarettes in a time interval of 15 min (sSm), while nSm (*n* = 10) were subjected to fresh air consumption for the same time span. Measurements were done in blood plasma samples taken 4 h after smoking using RRS. Data relative to the basic level (time 0 h) are illustrated by boxplot chart with percentiles 25 and 75, median (black line), mean (black square) and outliers (black cross), * *p* ≤ 0.05 to 0 h.

The analysis of the carotenoid level of β-carotene and lycopene in blood plasma of nSm and sSm (Figure 8) showed a significant reduction for the lycopene concentration in sSm 4 h after cigarette consumption; for β-carotene, no changes to the basic values were detected.

**Figure 9:**
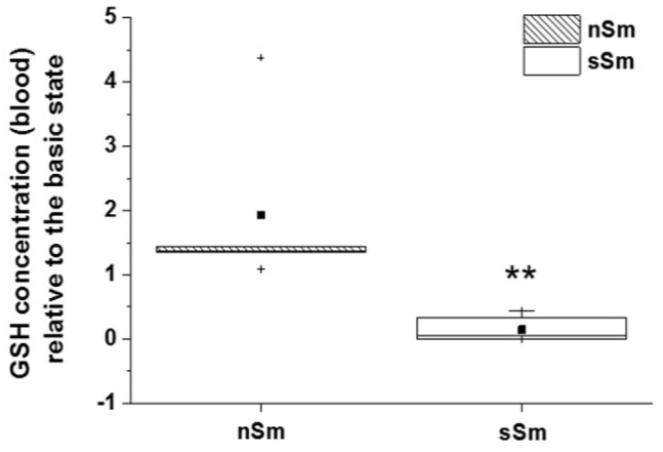
Impact of defined cigarette consumption on blood plasma levels of the endogenous AO GSH. sSm (*n* = 10) were allowed to smoke two cigarettes in a time interval of 15 min (sSm), while nSm (*n* = 10) were subjected to fresh air consumption for the same time span. Measurements were done in blood plasma samples taken 4 h after smoking using a fluorimetric assay. Data relative to the basic level (time 0 h) are illustrated by boxplot chart with percentiles 25 and 75, median (black line), mean (black square) and outliers (black cross), ** *p* ≤ 0.01.

While the GSH concentration in blood plasma remained stable for nSm 4 h after determination of the basic status (Figure 9), the GSH concentration of sSm dropped significantly 4 h after cigarette consumption by a factor of approximately 10 to the initial value. These data indicate an active consumption of the endogenous AO GSH to counter oxidative stress induced by tobacco consumption.

**Figure 10:**
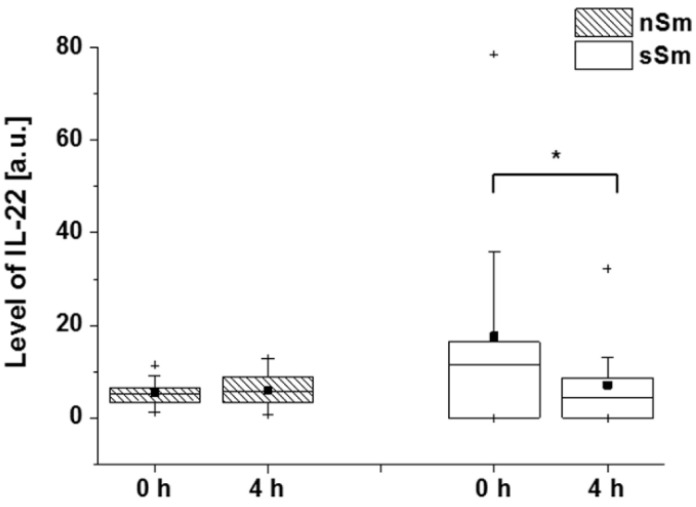
Impact of defined cigarette consumption on blood plasma levels of IL-22. sSm (*n* = 10) were allowed to smoke two cigarettes in a time interval of 15 min (sSm), while nSm (*n* = 10) were subjected to fresh air consumption for the same time span. Measurements were done in blood serum samples taken before (0 h) and 4 h after smoking by ELISA. Absolute data are illustrated by boxplot chart with percentiles 25 and 75, median (black line), mean (black square) and outliers (black cross), * *p* ≤ 0.05.

We also assessed the levels of IL-22 in smokers 4h after defined smoking. Interestingly, there was a clear drop of the primarily elevated levels of this mediator already, after that short time (Figure 10).

**Figure 11:**
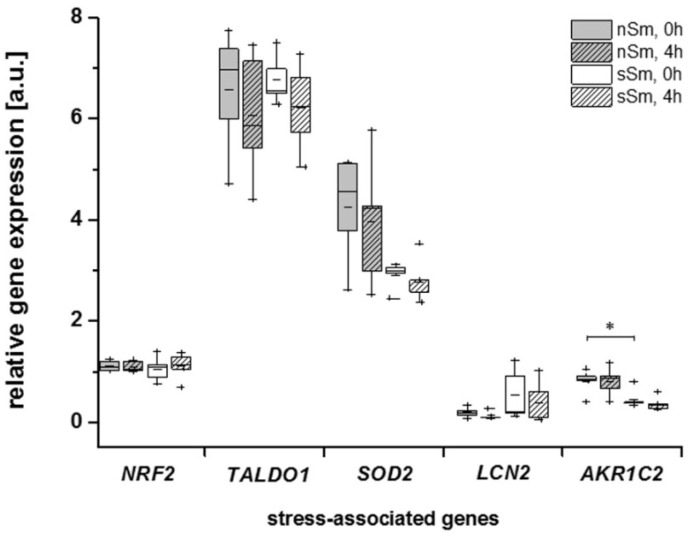
Comparison of the relative expression profile [a.u.] of oxidative stress-associated genes nuclear factor, erythroid 2 like 2 (NRF2/NFE2L2), transaldolase 1 (TALDO1), superoxide dismutase 2 (SOD2), lipocalin 2 (LCN2), aldo-keto reductase family 1, member C2 (AKR1C2)) by qRT-PCR analysis of nSm and sSm each before (0 h) and after (4 h) defined cigarette/fresh air consumption in blood mononuclear cells. Data are shown as expression of house-keeping gene hypoxanthine-guanine phosphoribosyltransferase (HPRT), presented by boxplot chart with percentiles 25 and 75, median (black line), mean (black square) and outliers (black cross), * *p* ≤ 0.05.

In a pilot study, we asked for the long-term and short-term impact of smoking on the expression levels of molecules associated with oxidative stress in blood immune cells. For this purpose, their basic levels, as well as their levels 4 h after cigarette/air consumption, were analyzed in a subgroup of five nSm and five sSm. Peripheral blood mononuclear cells showed strong expression of NRF2, SOD2 and TALDO1. With the exception of AKR1C2, which was downregulated in sSm, none of the assessed genes showed a significant decrease between sSm and nSm (Figure 11). LCN2 showed a trend to upregulation in sSm vs. nSm without reaching significance. These data exclude major oxidative stress accumulation in the blood cells of sSm, supporting our hypothesis of long-term compensatory adaptation of the AO status to permanent cigarette consumption. Defined cigarette consumption by sSm did not induce any relevant changes in gene expressions after 4 h (Figure 11). Notably, when considering all data from these substudy patients, the gene expression levels of AKR1C2 showed a trend to a negative correlation with blood IL-22 levels (Spearman’s correlation coefficient rs = 0.63, *p* = 0.062).

## 4. Discussion

A comparison of the cutaneous basic AO status between nSm and Sm revealed significant differences between these two groups (Figure 3 and Figure 4). RRS investigations demonstrated lower β-carotene and lycopene levels (exogenous AOs) in comparison to nSm (Figure 4). The lower total carotenoid values for smokers in skin are well known from previous studies, and were also found in the blood of our volunteers (Figure 6). The reduction is the result of a higher consumption of these AOs, due to the higher smoking-induced radical load. Several former investigations demonstrated that cutaneous carotenoids, which belong to lipophilic exogenous AOs, can be considered as marker substances for the body’s AO capacity, provided that no short-term changes in the lifestyle occur and the diet is balanced [13,35]. In contrast to the exogenous AOs, a closer look at the endogenous AOs in skin showed a significant increase in the basic GSH concentration for Sm versus nSm (Figure 3B). The determination of GSH from the uppermost layers of the skin (stratum corneum) does not show a snapshot, but a picture of the last ~30 days, and reflects the increased stress level in smokers [36,37]. GSH is ubiquitously present in cells and may be a first-line AO to protect against free radical damage [38]. This increased concentration indicates that the skin is adapting itself to the elevated radical amounts in these people [39,40], a phenomenon also observed in the directly exposed lung epithelium of Sm [41,42].

Furthermore, the detected increased cutaneous radical scavenging capacity in Sm, as detected by EPR as TEMPO decrease, even suggests a higher general AO status of the skin. This was surprising, as this behavior was detected so far only after AO supplementation [23]. In these former studies, carotenoids levels increased in parallel or remained stable, depending on the AO administered. In contrast to these studies, our data illustrate a more differentiated picture, as cutaneous carotenoid levels and total radical scavenging activity were opposed. The EPR technology, which is a non-invasive method, has been established and expanded as an effective method for the determination of the redox status in skin [23] and in cell culture [43]. TEMPO can lose its EPR visibility by reduction to the hydroxylamine by AOs, but also oxidation to the oxoammonium cation is possible [44], and has been observed in own investigation during the irradiation of skin cells [43,45].

In the case of TEMPO reduction, the EPR procedure does not discriminate between single substances, but detects the radical scavenging properties as a sum parameter, although the sensitivity towards different AO types apparently differs. While the spin probe TEMPO used in our study does not react with carotenoids [22], it does so with vitamin C. Our findings suggest that the enhanced endogenous AO defense system (in our study represented by GSH) could explain the unexpected high rate constants of the TEMPO decline in skin of the Sm (Figure 3A). This assumption was further supported by the comparable 1.5-fold increase in skin in both GSH levels and rate constant in Sm. The amphiphilic nature of TEMPO allows this probe to penetrate into cells, where it can interact with endogenous AOs [22]. However, we know from *ex vivo* investigations using the x-band EPR technique that approximately 50% of TEMPO is located in the stratum corneum [46], where the GSH was probed in the presented study. Furthermore, Fuchs J et al. have shown that the TEMPO decline in skin is related to reduction by AOs, because re-oxidation using the one-electron oxidizing agent potassium ferricyanide resulted again in a 90% increase of the EPR-visible nitroxide [47]. This leads to the assumption that the TEMPO decline in the skin could be mainly affected by local endogenous AOs. Nevertheless, reaction exogenous antioxidants, like vitamin C, cannot be excluded.

As described above, the surprising cutaneous increase in endogenous AOs may suggest an adaptation effect in the Sm. This result was specific to the skin compartment, but not clearly obvious in the blood of our Sm, which may be explained by the high fluctuation of all compounds in this compartment. Bizoń A et al., however, did find that Sm (≥20 cigarettes/day had increased blood concentrations of GSH, while nSm and mSm (<20 cigarettes/day) showed comparable blood GSH concentrations [48]. Another study supported the lacking effect of smoking of <20 cigarettes/day on blood GSH concentration [49]. In our study, we did not assess blood GSH concentrations in mSm.

When investigating the short-term effect (4 h) of controlled cigarette consumption, neither carotenoid levels, nor the TEMPO decline rate constant in skin, showed further changes (Figure 7) (GSH not assessed). This is in line with previous studies, demonstrating that AO supplementation leads not immediately to an increased cutaneous rate constant, but only after a time period of 2 to 8 weeks [23]. In fact, the skin has a reservoir function for substances that are either topically applied, administered systemically or synthesized by the body itself [50,51]. In contrast to the skin compartment, we observed a significant decline of lycopene (Figure 8) and GSH (Figure 9) blood levels in Sm 4 h after cigarette smoking.

In contrast to blood lycopene levels, β-carotene levels (which were already decreased in sSm; Figure 6) did not further decrease. Among the carotenoids, lycopene is the most effective quencher for singlet oxygen. Its quenching constant is about twice as high as that of β-carotene, and ten times higher than that of α-tocopherol [52]. The adaptation of the skin to long-term smoking by enhancing the endogenous AO levels might lower the deleterious effects of smoking. Considering the different functions of the exogenous AOs, this adaptation will not be sufficient to completely compensate their drop in smokers, as they have different functions and activities. Exogenous AOs have to be continuously supplemented by nutrition; the transport into skin can occur fast (vitamin C by active transporters) or takes hours to days (for lipophilic compounds like carotenoids or vitamin E, the latter reaches the skin via sweat and sebum) [53]. It should also be noted, however, that local endogenous AO upregulation cannot prevent premature skin aging in Sm, as frequently described [54,55].

Nevertheless, the Alpha-Tocopherol, Beta-Carotene Cancer Prevention (ATBC) Study was conducted in Finland as a joint project between the National Institute for Health and Welfare of Finland and the US National Cancer Institute (NCI), and could demonstrate that an excessive dosage of vitamins (exogenous AOs) can have a contrary effect on the metabolism. Male smokers, who were supplemented with high doses of vitamin E and ß-carotene, showed no protection against the development of lung cancer—conversely, the tumor rate rose quite unexpectedly [56,57]. An overview study of 2008 with various AOs resulted in an increased mortality rate for the supplementation of the vitamin A, whereas vitamin E supplementation had no effect [58]. Taken together, these studies raise the probability that a supplementation with AOs may have harmful as well as beneficial effects to counteract an imbalance in the AO homeostasis.

The study of a stress maker, for example for lipid peroxidation (especially 4-hydroxynonenal (HNE)), could provide further insight into the stress levels between non-smokers and smokers.

When assessing the effect of the basic AO status and controlled smoking in sSm at the level of the expression of molecules known to be induced by oxidative stress in blood immune cells, most of the assessed genes showed no significant changes (Figure 11). The only difference was found for AKR1C2, whose basic level was interestingly even downregulated in sSm. These data exclude major systemic oxidative stress in sSm, but support the long-term compensatory adaptation to permanent smoking.

AKR1C2 encodes an aldo-ketoreductase family 1 member, and has been shown to be induced upon acute activation (e.g., by oxidative stress) of the master transcription factor NRF2. The aldo-ketoreductase family comprises a group of are NAD(P)H-dependent oxidoreductases that are involved in the metabolism of xenobiotics derived, e.g., from tobacco, the detoxification of reactive lipid peroxides and aldehydes and in the synthesis of AOs, and cell protective substances like retinoic acid [59]. It should be noted that, in contrast to blood immune cells, AKR genes have been shown to be upregulated in the directly exposed airway epithelium of smokers [60]. To our knowledge, no previous data exist about AKR1C2 in immune cells in general and in smokers. Whether the selective downregulation of immune cell AKR1C2 expression and its possible negative association with IL-22 levels (Figure 10) reflects not only a balanced long-term oxidative state, but the suitability of AKR1C2 as an indicator of the body’s AO status, needs to be investigated in further, much broader studies, which should also include skin samples of Sm and nSm.

Interleukin-22 is a mediator previously shown to be elevated in blood of smokers [33], a trend which, together with a AKR1C2 level correlation, could also be demonstrated in our study participants (data not shown). More strikingly, controlled smoking induced a rapid (4 h) drop of systemic interleukin-22 in our study (Figure 10). Interleukin-22 is produced by activated specific T cells and innate lymphoid cells in tissues [34]. The function of this protective cytokine includes antimicrobial, regenerative and anti-oxidative actions on epithelia and liver [61,62,63]. Unfortunately, the tissue source and regulatory mechanisms of interleukin-22 production in smokers are not known so far. One may speculate that the short-term interleukin-22 drop results from smoking-induced acute oxidative stress in lung epithelial tissue through its influence on local interleukin-22 producers; however, this needs to be investigated in further, more mechanistic studies.

## 5. Conclusions

Based on our current knowledge, the following scenario can be proposed: the consumption of tobacco promotes an increase in the stress-level, promoting a stronger imbalance in the redox homeostasis for smokers vs. non-smokers. To counteract such processes, AOs from the blood are initially consumed. In order to be able to resist permanent stress situations by smoking, the metabolism produces more endogenous AOs. Their depletion in skin is time-delayed, whereby the skin displays a good reservoir for endogenous AOs, but also for those which were ingested with food. To protect the metabolism against oxidative stress induced by acute stress situations, the body is able to upregulate to a certain extent the endogenous AOs in a timely manner. AOs fluctuate in the blood and are mainly stored in the skin.

## Figures and Tables

**Table 1:** Volunteers’ characteristics and analyzed parameters assigned to the different studies (A–D).

Sub-Study	Smoking Status	Number of Participants	Gender: Female/Male	Analyzed Parameters
A	nSm	57	33/24	Basic (0 h) AO status in skin: radical scavenging capacity, carotenoids
	mSm	18	7/11	
	sSm	23	9/14	
B	nSm	13	9/4	Basic GSH (0 h) status in skin
	mSm	0	n.a.	
	sSm	13	5/8	
C	nSm	10	5/5	AO status 4 h after defined cigarette consumption compared to basis (0 h) status in skin and blood plasma of sSm: scavenging capacity, carotenoids, GSH and IL-22 (blood only)
	mSm	0	n.a.	
	sSm	10	5/5	
D	nSm ^#^	5	4/1	Basic status of expression of oxidative stress-associated genes in blood cells and its comparison to the status 4 h after defined cigarette consumption in sSm
	mSm	0	n.a.	
	sSm ^#^	5	3/2	

nSm, non-smokers; mSm, moderate smokers; sSm, strong smokers; ^#^ The results of substudy D are derived from the non-smoking-/smoking collective from substudy C; n.a.: not applicable.
